# In vitro influence of stem surface finish and mantle conformity on pressure generation in cemented hip arthroplasty

**DOI:** 10.3109/17453670902947382

**Published:** 2009-04-01

**Authors:** Gavin E Bartlett, Harinderjit S Gill, David W Murray, David J Beard

**Affiliations:** ^1^OOEC Nuffield Department of Orthopaedic Surgery, University of OxfordOxfordUK; ^2^Nuffield Orthopaedic CentreOxfordUK

## Abstract

**Background and purpose** Under physiological loads, debonded cemented femoral stems have been shown to move within their cement mantle and generate a fluid pump that may facilitate peri-prosthetic osteolysis by pressurizing fluid and circulating wear debris. The long-term physiological loading of rough and polished tapered stems in vitro has shown differences in performance, with greater interface pressures generated by the rough stems. In this study we investigated the individual effects of stem surface finish, degree of mantle wear, and mode of loading on the stem pump mechanism.

**Method** Rough and polished stems were loaded under different regimes in artificially worn cement mantles that permitted either 2 or 5 degrees of rotational stem movement, and the interface pressures were compared.

**Results** The pressures generated by the rough and polished stems were similar in either type of mantle. The pattern of pressure generation in the 2-degree mantles was similar to the pressures generated by rough stems after long-term loading, but the high posterior wall pressures fell and the tip pressures increased in the 5-degree mantles. The torsional loads were principal drivers of pressure generation in all areas of the interface other than the implant tip, where axial loading predominated.

**Interpretation** Femoral stems with rotational instability under cyclic torsional loads generate elevated interface fluid pressures and flows independently of stem surface finish. The rough surface finish is only important in creating this instability in tapered stems.

## Introduction

Periprosthetic osteolysis is the principal cause of long-term failure of cemented implants, and the femoral stem pump is possibly an important mechanism in facilitating this process (Malchau et al. 2002, Gregg et al. 2004, Bartlett et al. 2008). We have already partially characterized the mechanism using a model of a double-tapered Exeter stem (Stryker, Newbury, UK) with either polished or rough surface finish (Bartlett et al. 2009). The surface finish had no statistically significant effect on the pressures generated by stems within mantles that fully conformed to the stem. However, only the stems with a rough surface finish developed rotational instability as a result of long-term, repetitive physiological loads and greater interface pressures under physiological loads. The same loading schedule did not cause a statistically significant change in the interface pressures generated by the polished stems (Bartlett et al. 2009).

The enhanced pumping effect of the rough stem was therefore related to a combination of the stem surface finish, the combined axial and torsional loads applied to the stem, and the resultant increased rotational movement of the stem, but the relative importance of these factors is not known. The effect of further increases in rotational movement of the stem beyond that generated in the study is also not known. Our hypotheses in this study were that (1) the pressures generated under physiological loads are directly proportional to the rotational micro-motion of the stem within the cement mantle and are therefore also dependent on the torsional forces applied to the stem; and (2) the surface finish of the prosthesis continues to influence the fluid pressures generated in worn mantles, as seen at the interface of conforming mantles (Bartlett et al. 2009).

## Method

The study was performed using the Hip Arthroplasty Pressure Simulator (HAPS), previously reported as a reliable and reproducible in vitro model of stem pumping (Bartlett et al. 2008).

We assessed the pressures generated in vitro by 2 groups of model Exeter stems (Stryker, Newbury, UK) with either rough or polished surface finishes, loaded within 2 groups of worn cement mantles that were created artificially. The first group of 3 cement mantles permitted 2 degrees of rotational movement; this was roughly double the movement that was recorded in cement mantles in which rough stems were cyclically loaded over 1 million cycles (mean 0.8°). The second group of 3 mantles permitted a further increase in rotational micromotion of 5 degrees. Thus, the mantles represent the result of further cement mantle wear beyond that performed in the previous study (Bartlett et al. 2009).

**Figure 1.**
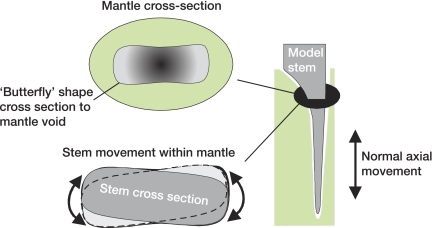
Schematic diagram of the mantle void profile created by the formers, demonstrating the increased rotational movement and unchanged axial movement of the stem.

We used the same design of scaled model stems, based on a 37.5 mm offset size 1 Exeter stem (Stryker) as previously reported (Bartlett et al. 2008, 2009). The surface finishes of the stems were also prepared in a similar way by the manufacturers of the Exeter stem (Stryker Orthopaedics, Hérouville Saint Clair, France) and either polished to tolerances of the current Exeter stem (0.03 µm Ra) or shot-blasted to create surface roughness in excess of 2 µm Ra.

The worn cement mantles were created using specially designed formers. The profile of each former was created by manipulation of the CAD file of an Exeter size 1, 37.5 stem offset stem supplied by the manufacturer (Stryker). The shapes of formers were created using a geometric engineering package (Patran; MSC Software Corporation, Santa Ana, CA); the stem was rotated around its longitudinal axis by either 2 or 5 degrees and its images at each extreme of rotational movement were combined to produce a former with a “butterfly” cross section (Figure 1). The formers thus created a void in the cement, in which the standard model stems could rotate around their longitudinal axes by either 2 or 5 degrees. The formers were manufactured in aluminium from their computer-generated designs. The cementation process followed the same method as previously described, except that the mantle void was created using one of the formers.

**Figure 2.**
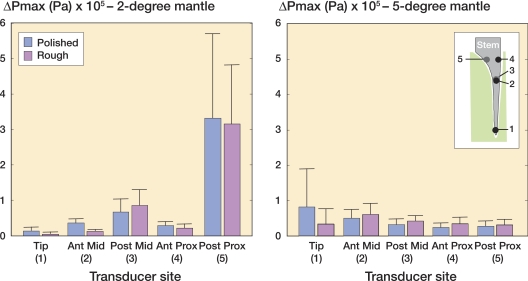
ΔPmax values (in Pa) recorded at each transducer site (see insert) for both rough and polished stems under combined physiological loading, in both the 2-degree and 5-degree mantles.

The same HAPS apparatus was used as previously described (Bartlett et al. 2009); this provided a central testing chamber at 37°C containing a cement mantle and model stem immersed in a synthetic synovial fluid (vegetable oil). The pressure at the stem/cement interface was sampled by 5 external transducers communicating with the interface via pressure tappings that traversed the wall of the chamber and the cement mantle at different locations (Figure 2, insert).

The same combined loading schedule was applied to the HAPS chamber lid by a materials-testing machine (MTM) along 2 perpendicular axes (Bartlett et al. 2009). The axial, longitudinal forces were applied in a sinusoidal pattern oscillating between 1.3 kN and 0.2 kN at a frequency of 1 Hz. The torsional forces were applied perpendicular to the longitudinal axis of the stem, in a stepped pattern coordinated to the axial load. The retroverting force of 8 Nm was greater than the anteverting force of 0.46 Nm and occurred during the positive axial load ramp; this switched to the anteverting force during the negative axial load ramp. The axial and torsional loads were also applied in isolation at the same magnitude and cycle frequency as during combined loading.

Three 2-degree mantles were assessed with both rough and polished stems under 3 different loading regimes: isolated axial and torsional loading, and combined torsional and axial loads. We performed 3 trials for every loading condition; each trial was separated by disassembly and reassembly of the apparatus and consisted of 500 initial load cycles to bed in the stem followed by a data sampling period of 30 load cycles. Three 5-degree mantles were then assessed with both stem types using the same method, but only under combined torsional and axial loading conditions.

For each load cycle measured, the magnitudes of the positive and negative pressure peaks were identified to establish the maximum amplitude of the pressure wave at each transducer site. This value, ΔPmax (expressed in pascals), was the primary variable in this study.

### Statistics

All data were processed using custom-written Matlab routines (version 7; Mathworks, Inc., Natick, MA). The data were not normally distributed; therefore, a median ΔPmax value represents the 3 trials sampled for each mantle. As there were only 3 mantles in each study group, however, a mean value and standard deviation were used to describe the distribution of median ΔPmax values for each transducer site. A Wilcoxon sign rank test was performed on the paired median ΔPmax values of all 5 transducer sites (n = 15 in each study group) for either the rough or polished stems under different loading regimens within the three 2-degree mantles. A Wilcoxon rank sum test was performed on the unpaired data of the 2- and 5-degree mantles with either polished or rough stems in situ (n = 15 in each group). All statistical analyses were performed using Matlab v.7 and statistical significance was set at α ≤ 0.05.

## Results

The artificially worn cement mantles permitted reproducible movements of the model stems under physiological loading. The mean axial recoverable micromovements for both the 2- and the 5-degree mantles were approximately 0.35 mm for both stem types. The mean rotational micromovements of the polished stem accurately represented the range of motion intended by the former design; the rough stems rotated through reduced mean arcs of 1.42° and 3.17°, respectively (Table).

**Table:** Axial micromotion (in mm) and torsional micromotion (in degrees) of rough and polished stems in the 2- and 5-degree artificially worn mantles

	2-degree mantles				5-degree mantles			
Surface finish	Polished		Rough		Polished		Rough	
	Mean	SD	Mean	SD	Mean	SD	Mean	SD
Axial (mm)	0.35	< 0.01	0.37	< 0.01	0.38	0.04	0.35	0.02
Rotational (degrees)	2.12	0.54	1.42	0.39	4.84	0.47	3.17	0.89

SD: standard deviation.

The surface finish of the stem had no statistically signifi-cant effect on the interface pressures generated in either the 2-degree mantle (p = 0.3) or the 5-degree mantle (p = 1) (Figure 2).

**Figure 3.**
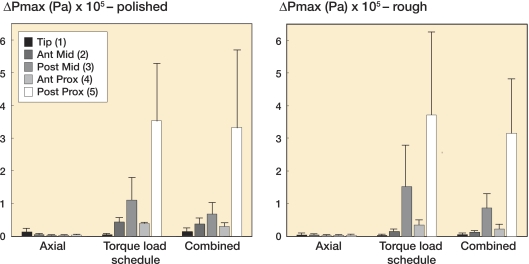
ΔPmax values (in Pa) for rough and polished stems within artificially created 2-degree mantles at each pressure transducer site (see insert). The ΔPmax values were recorded under 3 different loading regimens: isolated axial loading, isolated torsional loading, and combined physiological loading.

The principle differences in ΔPmax values between the 2- and 5-degree mantles occurred at transducer sites 1, 3, and 5. The high pressures generated on the posterior wall of the 2-degree mantles (sites 3 and 5; Figure 2), which reached a maximum mean ΔPmax of 330,000 Pa, were not reproduced in the 5-degree mantles. The pressure levels recorded on the posterior wall of the 5-degree mantles matched the levels on the anterior wall (mean 32,000 Pa). However, the pressures at both polished and rough implant tips (site 1) in the 5-degree mantles were higher than the pressures in the 2-degree mantles (mean 63,000 Pa in the 5-degree mantles as opposed to 13,000 Pa in the 2-degree mantles). These observed differences in pressure generation over all 5 transducer sites between the 2- and 5-degree mantles did not reach statistical significance for either the rough or the polished stems (p ≥ 0.4). The magnitude of pressures generated by both rough and polished stems in the 2-degree mantles was highly dependent on the type of load applied (Figure 3). There was no statistically significant difference between isolated torsional loads and combined axial and torsional loads for either type of stem (p ≥ 0.2). The isolated torsional loads closely replicated the distribution of pressures generated by combined axial and torsional loading at all transducer sites except at the implant tip (site 1). The pressures generated overall by isolated axial loads were less than either torsional or combined loads (p < 0.001), but isolated axial loading at the tip generated greater average ΔPmax values than torsional loading, which were equivalent to pressures generated by combined loads.

## Discussion

The purpose of this study was to characterize the femoral stem pump mechanism further by assessing (1) the effects of further increases in rotational stem micromotion, (2) the importance of the torsional load that creates this movement, and (3) the influence of stem finish.

The stem micromovements recorded in this study show that the artificially worn cement mantles permitted rotational micromotion of the polished stems by either 2 or 5 degrees, with constant amounts of axial movement. The reduced rotation of the rough stems in the mantles may be related to increased frictional forces between the walls of the mantle and the stem compared to the polished stems. Interestingly, the reduced movements of the rough stems did not translate into a reduction in ΔPmax values at the interface compared to the polished stems. This pattern of stem micromotion is an extension of the pattern of stem motion observed after long-term, cyclic physiological loading of rough stems, and was intended to represent the result of further cement mantle wear from extended long-term loading beyond that performed in the previous study (Bartlett et al. 2009).

The pattern and magnitude of ΔPmax generated in the 2-degree mantles by both rough and polished stems is closely related to the mean pressures generated by rough stems after long-term physiological loading. Thus, the increased range of rotational stem movement of 2 degrees had no enhancing effect on the pump mechanism. Further increases in stem rotation to 5 degrees led to reductions in the high posterior wall pressures and to paradoxical increases in the low pressures at the tip leading to the equalization of pressures across the entire interface. This study therefore demonstrates that the peak pressures generated by the stem pump occur relatively early in the process of cement mantle wear by the stem. By 5 degrees of stem movement, when the stem is visibly loose, the high posterior wall pressures have fallen, though pressures still remain sufficiently high (35,000 Pa or greater) to induce macrophage differentiation, a key step in osteolysis in aseptic loosening (McEvoy et al. 2002). Furthermore, although the peak pressures are less, the volumes of pressurized interface fluid in the 5-degree mantles have increased and this may have a significant clinical impact on periprothetic tissue, as the increased volumes of fluid will be less easily accommodated by changes in tissue compliance.

Analysis of the pressure waveforms generated by combined loading of stems in worn cement mantles suggested that the pressure peaks in each cycle were generated by the torsional load (Bartlett et al. 2008). This finding was verified by the statistically significant difference in pressures generated by isolated torsional and axial loading of the stem within the 2-degree mantles. The similar performances of both polished and rough stems within the artificially worn cement mantles indicated that the different behaviors of the 2 stem surface finishes during long-term loading were due to the fact that the rough stems were causing asymmetric deformation of the cement mantle and increased rotational movement—and not due to any influence on interface fluid dynamics. This therefore suggests that defective cement mantles that permit polished stems to develop rotational instability would also experience a similar augmentation of the pump mechanism, which emphasizes the importance of sound surgical cementing techniques.

Our findings contribute to our understanding of aseptic loosening of cemented femoral stems secondary to peripros-thetic osteolysis. In vivo and in vitro modeling of the osteo-lytic process has identified elevated cyclical fluid pressure as a potent stimulator of macrophage/osteoclast differentiation and osteolysis (Skripitz and Aspenberg 2000, McEvoy et al. 2002, Skoglund and Aspenberg 2003). There is indirect clinical evidence of pressure generation within failing cemented hip replacements but whether this also occurs in asymptomatic, well-fixed joints has not been shown (Hendrix et al. 1983, Robertsson et al. 1997). It has been proposed that pressure generation may be linked to prosthetic movement (Anthony et al. 1990). Dynamically induced micromotion has been measured in newly implanted, cemented stems and clinical retrieval studies have provided microscopic evidence of stem surface wear secondary to micromotion of the stem with respect to the cement (Glyn-Jones et al. 2004, Howell et al. 2004). Our observations provide evidence that this level of stem movement is sufficient to generate fluid flow and pressure at the stem-cement interface of newly implanted stems, and to create a pumping mechanism—albeit of small volumes of fluid. If no change in stem recoverable micromotion occurs, then this pumping mechanism remains unchanged, acting on small volumes of fluid that are unlikely to substantially increase the pressure if exposed to compliant biological structures. This is consistent with the excellent clinical results with the polished Exeter stem (Williams et al. 2002). However, consistent wear patterns created by rotational micromotion were identified on the surfaces of stems that were revised for aseptic loosening (Howell et al. 2004). We have shown that increased rotational micromotion increases interface pressures that also act on larger volumes of interface fluid. Any possible defects in the cement mantle will expose the adjacent subchondral bone to the elevated cyclical fluid pressure and to particles suspended in the fluid. This provides a mechanism of aseptic stem loosening and an explanation for the different long-term survival rates reported for stems of identical geometry but with matt or polished surface finishes (Collis and Mohler 2002, Malchau et al. 2002).
